# *Nicotiana benthamiana* Methanol-Inducible Gene (MIG) 21 Encodes a Nucleolus-Localized Protein That Stimulates Viral Intercellular Transport and Downregulates Nuclear Import

**DOI:** 10.3390/plants13020279

**Published:** 2024-01-17

**Authors:** Ekaterina V. Sheshukova, Kamila A. Kamarova, Natalia M. Ershova, Tatiana V. Komarova

**Affiliations:** 1 Vavilov Institute of General Genetics, Russian Academy of Sciences, 119991 Moscow, Russia; sheshukova@vigg.ru (E.V.S.); kamila.kamarova@vigg.ru (K.A.K.); ershova@vigg.ru (N.M.E.); 2 Belozersky Institute of Physico-Chemical Biology, Lomonosov Moscow State University, 119991 Moscow, Russia

**Keywords:** methanol, methanol-inducible gene, MIG21, viral intercellular transport, nuclear import, inducible promoter, plant resistance, tobacco mosaic virus

## Abstract

The mechanical damage of plant tissues leads to the activation of methanol production and its release into the atmosphere. The gaseous methanol or vapors emitted by the damaged plant induce resistance in neighboring intact plants to bacterial pathogens but create favorable conditions for viral infection spread. Among the *Nicotiana benthamiana* methanol-inducible genes (MIGs), most are associated with plant defense and intercellular transport. Here, we characterize *NbMIG21*, which encodes a 209 aa protein (NbMIG21p) that does not share any homology with annotated proteins. NbMIG21p was demonstrated to contain a nucleolus localization signal (NoLS). Colocalization studies with fibrillarin and coilin, nucleolus and Cajal body marker proteins, revealed that NbMIG21p is distributed among these subnuclear structures. Our results show that recombinant NbMIG21 possesses DNA-binding properties. Similar to a gaseous methanol effect, an increased *NbMIG21* expression leads to downregulation of the nuclear import of proteins with nuclear localization signals (NLSs), as was demonstrated with the GFP-NLS model protein. Moreover, upregulated *NbMIG21* expression facilitates tobacco mosaic virus (TMV) intercellular transport and reproduction. We identified an *NbMIG21* promoter (PrMIG21) and showed that it is methanol sensitive; thus, the induction of *NbMIG21* mRNA accumulation occurs at the level of transcription. Our findings suggest that methanol-activated *NbMIG21* might participate in creating favorable conditions for viral reproduction and spread.

## 1. Introduction

Due to an attached lifestyle, plants are constantly exposed to abiotic (drought, salinity, heat, cold, lack of nutrients, lack of light, and high light intensity) and biotic (viruses, microorganisms, insects, and herbivorous animals) environmental stressors [1]. During evolution, plants developed two main ways to coordinate their generalized defense reactions: intercellular communication via plasmodesmata (PD) and signal transfer by the emission and perception of volatile organic compounds (VOCs). PD serve for the transport of various regulatory molecules including RNA and proteins, in addition to low-molecular-weight compounds [2]. PD permeability is under strict control and is regulated via several main mechanisms: (i) physical narrowing of the channel by callose depositions mediated by callose-degrading and callose-synthesizing enzymes, (ii) regulation by various proteins associated with and localized in the PD, and (iii) signals from mitochondria and chloroplasts that are transferred to the nucleus and affect the expression of various genes that encode proteins, the ensemble of which defines the PD state [3,4,5]. Therefore, PD regulation usually involves the nucleus and nucleus-encoded genes. Pathogenic bacteria and viruses could interfere with a plant’s immune response, affecting the nucleus and nucleocytoplasmic transport as well as the PD and symplastic transport.

To realize the intra- and interplant communication in the absence of physical contact, plants utilize VOCs [6,7]. In addition to simple molecules such as oxygen, carbon dioxide, and water vapor, plants emit a huge amount of various complex compounds such as terpenoids, derivatives of fatty acids and amino acids, benzoids, phenylpropanoids, etc., into the atmosphere [8]. Among them is gaseous methanol that is a product of cell wall pectin demethylation by pectin methylesterase (PME) [9,10]. Methanol is one of the signal molecules that is emitted both in normal conditions [11,12] and in response to stress [13,14,15]. It plays a significant role in plant–herbivore [16,17] and plant–pathogen interactions [18]. Methanol emitted by an injured plant triggers defense responses in both its own intact leaves and neighboring plants; it induces resistance to the bacterial pathogens *Agrobacterium tumefaciens* and *Ralstonia solanacearum* [18]. Moreover, it was shown that transgenic plants with *PME* overexpression are characterized by an elevated level of methanol emission [18] and resistance to polyphagous insect pests [19]. The analysis of the transcriptome of *PME*-transgenic tobacco plants with increased methanol emission revealed changes in the expressions of transcription factors related to plant–herbivore interactions together with cell wall-modifying enzymes [17]. The molecular mechanisms underlying the methanol-induced defense response are still mainly unknown. Methanol was demonstrated to activate specific factors, encoded by methanol-inducible genes (MIGs) which participate in the plant’s resistance to both abiotic and biotic stress factors [18,20]. Most of these genes are involved in defense reactions and intercellular transport. The treatment of plants with a physiological concentration of gaseous methanol leads to the activation of intercellular transport of the macromolecules and impeded nucleocytoplasmic transport that results in the creation of favorable conditions for a viral infection as an unintended consequence of the induced antibacterial resistance [18]. Among the selected MIGs are *NbAELP* (a gene encoding aldose-1-epimerase-like protein, previously designated NCAPP) and *beta-1,3-glucanase* (BG), which were shown to participate in the activation of intercellular transport of the macromolecules and the stimulation of tobacco mosaic virus (TMV) RNA accumulation [18]. Moreover, NbAELP was demonstrated to affect nucleocytoplasmic transport [21] as well as PME [22].

PD act as pathways for transporting different compounds such as proteins, nucleic acids, hormones, and metabolites, crucial for signaling during plant development and defense [5]. The flow through PD is controlled by various mechanisms, among which the most extensively studied is the callose-dependent regulation of PD permeability via the modulation of callose depositions in the cell wall near these channels. Callose turnover is maintained by several enzymes that are involved in callose synthesis, degradation, and stabilization [23]. Furthermore, research into the proteins associated with PD [24], the interactions between the endoplasmic reticulum and plasma membrane (PM) around these channels [25], and the specific lipid composition of the PM within them [26] has broadened our understanding of PD function [27]. Recent evidence suggests that signals from different cell organelles, primarily mitochondria and chloroplasts, also play a role in controlling PD. These signals are transmitted to the cell nucleus and impact the expression of genes involved in regulating intercellular transport and PD function [4,28,29]. However, the exact mechanisms and components involved in this signaling pathway are still to be elucidated. Therefore, PD regulation involves proteins within the channels themselves as well as signal pathways from organelles to the nucleus.

Previously, among the MIGs we have identified, a gene with an unknown function, designated *NbMIG21*, demonstrated its involvement in the regulation of the intercellular transport of macromolecules as well as its ability to stimulate TMV-based vector reproduction [18]. In the current study, we investigated *NbMIG21* and assessed its effect on nucleocytoplasmic transport, local TMV spread, and reproduction. We demonstrated that NbMIG21p has nuclear localization and is concentrated in subnuclear structures, in particular, the nucleolus. Similar to NbAELP and PME, NbMIG21p interferes with nucleocytoplasmic transport as was demonstrated using the GFP-NLS reporter molecule. Moreover, an increased *NbMIG21* expression stimulates the intercellular transport of the TMV-based viral vector. Finally, we have isolated the *NbMIG21* promoter region, demonstrated its sensitivity to methanol, and showed that recombinant NbMIG21p has DNA-binding properties, being able to bind various promoters including its own one.

Thus, we suggested that NbMIG21 could be one of the players participating in organelle-to-nucleus plasmodesma signaling, affecting both nucleocytoplasmic and intercellular transport. We hypothesize that NbMIG21 might participate in creating favorable conditions for the viral reproduction, (i) interfering with nucleocytoplasmic transport of the macromolecules that could include antiviral cellular factors and (ii) increasing PD permeability, leading to enhanced viral spread.

## 2. Results

### 2.1. Analysis of N. benthamiana Methanol-Inducible Gene 21 (NbMIG21)

**Figure 1:**
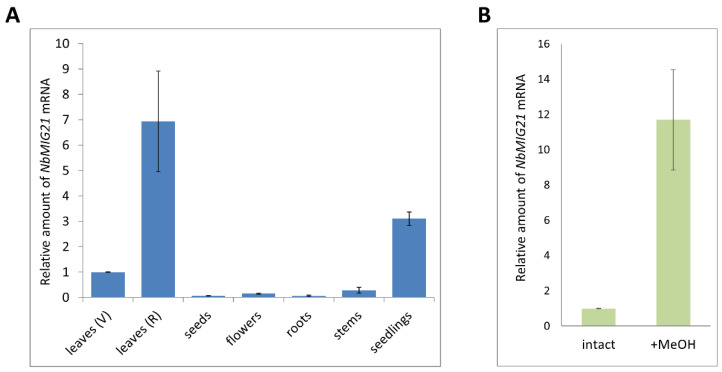
Quantitative RT-PCR analysis of *NbMIG21* mRNA levels in different parts of *N. benthamiana* plants (**A**) and in leaves upon methanol treatment (**B**). (**A**) Seedlings were grown for 10 days after germination. Leaves (V) were harvested from 3-week-old plants (vegetative stage); leaves (R), stems and flowers were harvested from same plants 5 weeks later (reproductive stage). Seeds were collected from 10-week-old plants. (**B**) Relative amount of *NbMIG21* mRNA isolated from leaves (V) of *N. benthamiana* plants incubated in a sealed desiccator with an elevated methanol (+MeOH) concentration for 18 h. The difference between samples from intact leaves and after methanol treatment is significant at *p* < 0.001 (Student’s *t*-test). The levels of expression for both (**A**) and (**B**) are normalized to the *PP2A* gene. The *NbMIG21* expression level in leaves (V) was taken as 1. The plot represents the mean ± SE of three technical repeats from three biological replicates each.

*Nicotiana benthamiana methanol-inducible gene 21* coding sequence (*NbMIG21*, GenBank Ac GU128961) was identified among genes expression of which increased in response to gaseous methanol or vapors from the wounded plant [18]. Initially *NbMIG21* was isolated from leaves. Here, we analyzed *NbMIG21* expression in different parts of the plant as well as in seedlings using quantitative real-time PCR (qRT-PCR) (Figure 1A). It appears that the leaves are characterized with the highest level of expression that is in line with the results based on transcriptome data from the *N. benthamiana* Gene Expression Atlas (http://sefapps02.qut.edu.au/atlas/tREX6.php, accessed on 21 January 2022) resource (Figure S1). Moreover, *NbMIG21* mRNA accumulation in leaves increases with their age.

To confirm that NbMIG21 is upregulated in response to methanol, we performed 18 h incubations of *N. benthamiana* plants in a desiccator with an elevated gaseous methanol concentration. The samples for mRNA analysis were collected before and after incubation from the leaves of the same plants. The qRT-PCR results demonstrated a significant upregulation in *NbMIG21* expression in response to the gaseous methanol treatment (Figure 1B).

### 2.2. NbMIG21 Promoter Identification and Verification

Since the *NbMIG21* mRNA level increases in response to the gaseous methanol treatment, its regulation could occur at the transcription level. We hypothesized that the *NbMIG21* promoter could be methanol sensitive. To check this assumption, we isolated a region of 1128 bp upstream of the *NbMIG21* coding sequence from *N. benthamiana* genomic DNA. It completely corresponded to the reference genome scaffold available at https://solgenomics.net/organism/Nicotiana_benthamiana/genome (accessed on 11 May 2022). The bioinformatic analysis of the obtained sequence using the PlantCARE tool (https://bioinformatics.psb.ugent.be/webtools/plantcare/html/, accessed on 15 May 2022) (Table S1) revealed elements involved in the response to light stress as well as motives related to the recognition of abscisic and salicylic acids.

To verify the ability of the isolated sequence (designated PrMIG21, ENA Ac. ON254805) to serve as a promoter and to direct mRNA synthesis, we generated the genetic construct PrMIG21-GFP containing a *GFP* reporter gene downstream of the putative promoter. PrMIG21-GFP was delivered into the cells of *N. benthamiana* leaves via agroinfiltration.

**Figure 2:**
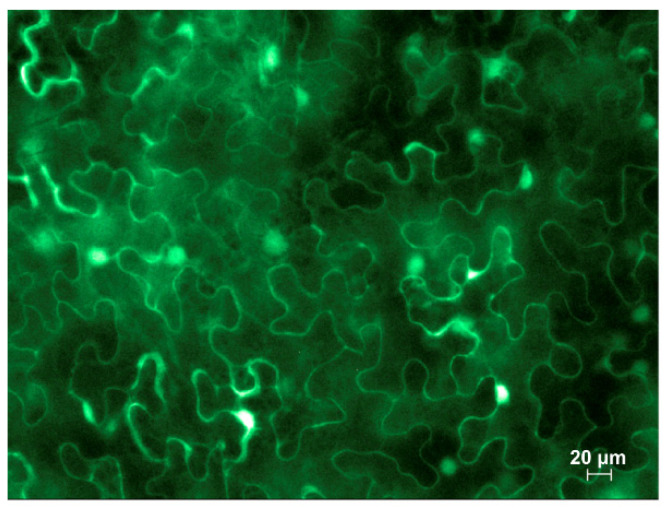
Fluorescent microscopy image of GFP accumulation in epidermal cells of leaves infiltrated with PrMIG21-GFP at 3 dpi. Mixture for infiltration was supplemented with agrobacteria containing plasmid for expression of p19 silencing suppressor of tomato bushy stunt virus.

Infiltrated leaves on the third day after infiltration were examined under a fluorescence microscope and GFP fluorescence was clearly observed (Figure 2), indicating that PrMIG21 is able to direct mRNA synthesis, and thus, possesses promoter activity.

### 2.3. PrMIG21 Is Methanol Sensitive

**Figure 3:**
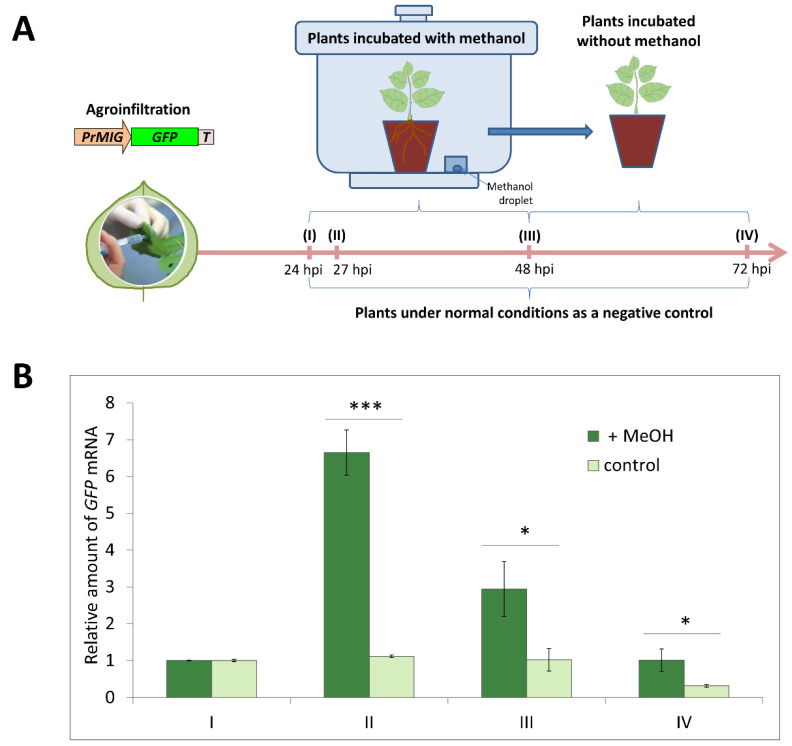
PrMIG21 is methanol sensitive. (**A**) Schematic representation of experimental workflow, with sample collecting time points indicated. (**B**) Relative amount of *GFP* mRNA in leaves of *N. benthamiana* plants agroinfiltrated with PrMIG21-GFP and treated with gaseous methanol. Amount of *GFP* mRNA at time point “I” is taken as 1. Student’s *t*-test was applied to assess statistical significance of difference between control plants and plants incubated with methanol. *: *p* < 0.05, ***: *p* < 0.001.

In order to assess if PrMIG21 is methanol sensitive and evaluate the effect of gaseous methanol on the efficiency of PrMIG21-directed *GFP* mRNA accumulation, we treated plants with a physiological concentration of methanol after infiltration and collected samples at different time points. The experimental setup and workflow is presented in Figure 3A. Fully expanded *N. benthamiana* leaves were infiltrated with *Agrobacterium* suspensions containing the PrMIG21-GFP construct. At 24 h post infiltration (hpi), leaf samples were collected with a hole punch, and then, one group of plants was placed for 24 h into the 20 L desiccator containing a droplet of methanol on filter paper (200 µL) to achieve a gaseous methanol concentration comparable with the physiological [18], and the other group (control) was incubated without methanol (Figure 3A). Then, all plants were kept under normal greenhouse conditions for another 24 h. The samples were collected at the following time points: 27 hpi (3 h with methanol), 48 hpi (24 h in methanol), 72 hpi (24 h after methanol treatment). The relative amount of *GFP* mRNA was determined using qRT-PCR (Figure 3B).

Our results show that the *GFP* mRNA level drastically increased after a 3 h incubation with gaseous methanol, with a further decrease at the next time point (at the end of 24-h methanol treatment) and recovering to the normal level after another 24 h under normal conditions. Thus, PrMIG21 is methanol sensitive and is rapidly activated in response to gaseous methanol within the first hours.

### 2.4. NbMIG21-Encoded Protein Sequence Analysis and Intracellular Localization

**Figure 4:**
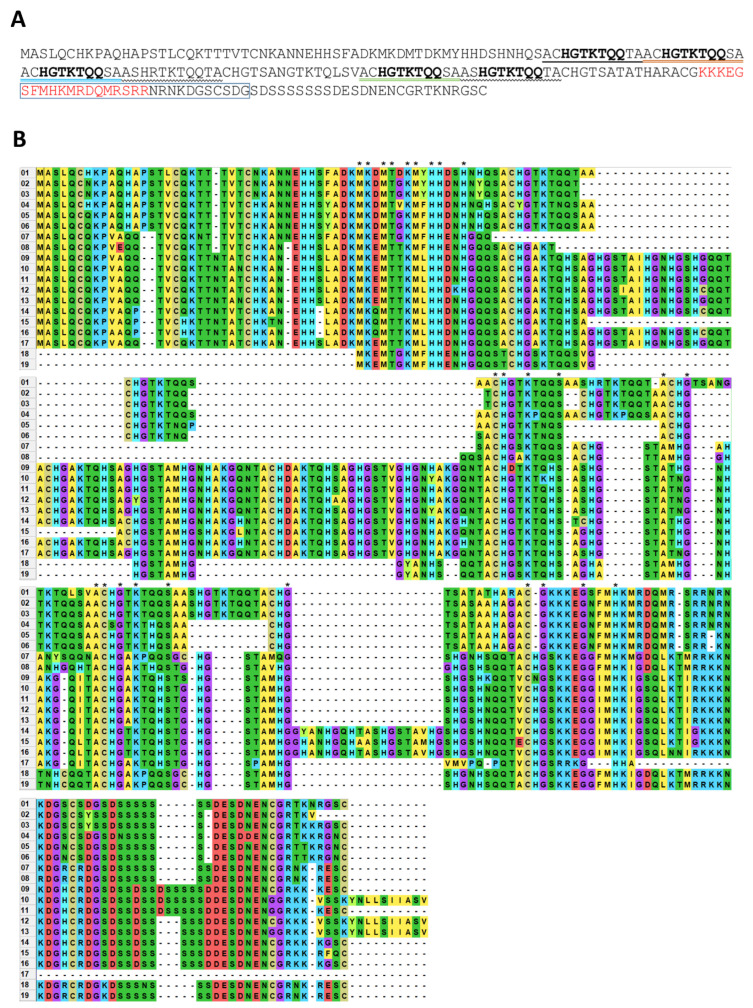
NbMIG21p sequence analysis. (**A**) NbMIG21p sequence. Five 7-amino-acid long repeats are in bold. 11-amino-acid stretches are underlined: colored double lines mark perfect repeats, single or waived—imperfect. Letters in red—NLS predicted by LOCALIZER (https://localizer.csiro.au/, accessed on 10 June 2022); letters in a box—NoLS predicted using NOD http://www.compbio.dundee.ac.uk/www-nod/index.jsp (accessed on 10 June 2022). (**B**) Multiple alignment of MIG21p homologues from *Solanaceae* species performed using MEGA 11 software [34] with ClustalW algorithm. Asterisk indicates conservative residues. 01—NbMIG21 (ACY74744.2), 02—*N. attenuata* hypothetical protein (OIT27799.1), 03—*N. attenuata* uncharacterized protein (XP_019232815.1), 04—*N. sylvestris* protein SSUH2 homolog (XP_009768095.1), 05—*N. tabacum* uncharacterized protein (XP_016494112.1), 06—*N. tomentosiformis* hornerin-like (XP_009592421.1), 07—*N. attenuata* uncharacterized protein (XP_019264939.1), 08—*D. stramonium* hypothetical protein (MCD7459440.1), 09—*S. stenotomum* uncharacterized protein (XP_049376514.1), 10—*S. verrucosum* hypothetical protein (WMV44005.1), 11—*S. tuberosum* hornerin-like (XP_006343871.1), 12—*S. commersonii* hypothetical protein (KAG5593335.1), 13—*S. verrucosum* uncharacterized protein (XP_049361851.1), 14—*S. lycopersicum* hornerin (XP_004245528.1), 15—*S. chilense* hypothetical protein (TMW85354.1), 16—*S. pennellii* hornerin-like (XP_015085049.1), 17—*S. tuberosum* hypothetical protein (CAA12355.1), 18—*N. tabacum* hornerin-like (XP_016480098.1), and 19—*N. sylvestris* hornerin-like (XP_009763094.1).

*NbMIG21*-encoded protein, NbMIG21p, contains five imperfect repeats of 11 amino acid (aa) residues (Figure 4A). NbMIG21p does not share any homology with annotated proteins in the UniProt database. A motif search service (https://www.genome.jp/tools/motif/, accessed on 28 April 2023) revealed that the NbMIG21p fragment from position 15 to 81 contains a motif corresponding to ubinuclein conserved middle domain (PF14075) [30] (Figure S2). Moreover, among the predicted and putative proteins, similar sequences were discovered in several *Solanaceae* species from the genera *Nicotiana*, *Solanum*, and *Datura* (Figure 4B). The NbMIG21p C-terminal sequence contains multiple positively charged lysine and arginine residues that are characteristic of a nuclear localization signal (NLS). NbMIG21p analysis using a bioinformatic tool for NLS prediction—LOCALIZER (https://localizer.csiro.au/, accessed on 10 June 2022) [31]—revealed an NLS, while a nucleolus localization signal (NoLS) predictor—NOD (http://www.compbio.dundee.ac.uk/www-nod/index.jsp, accessed on 10 June 2022) [32,33]—identified an NoLS in the NbMIG21p sequence.

**Figure 5:**
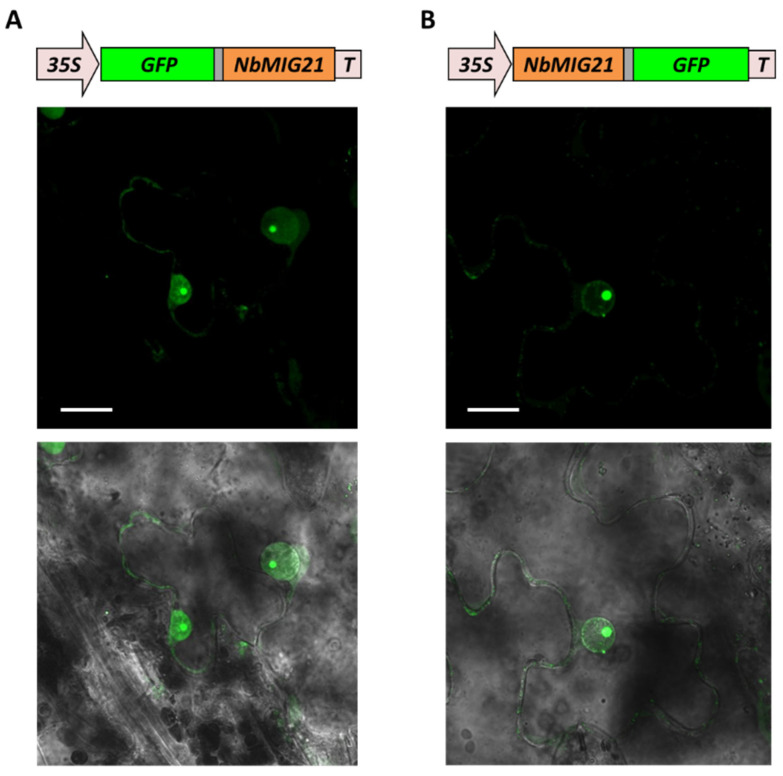
NbMIG21p intracellular localization. Images of 35S-GFP:NbMIG21 (**A**) or 35S-NbMIG21:GFP (**B**) expressing epidermal cells of *N. benthamiana* leaves at 3 dpi, obtained using confocal fluorescence microscopy. Projection of several confocal sections (top) superimposed on a bright field image of the same cell (bottom). Bars = 20 μm. 35S: cauliflower mosaic virus 35S promoter; T: 35S terminator of transcription.

To analyze NbMIG21p, subcellular localization genetic constructs encoding NbMIG21p and a GFP fuse were obtained: 35S-NbMIG21:GFP and 35S-GFP:NbMIG21. To exclude the reporter protein position effect, GFP was added to NbMIG21p as an N-terminal or C-terminal fuse. Moreover, the construct encoding the NbMIG21p fuse with RFP was also tested (Figure S3). All constructs were based on the pCambia1300 backbone supplemented with an additional 35S-promoter and terminator. *N. benthamiana* leaves were infiltrated with agrobacteria containing either a 35S-NbMIG21:GFP, 35S-GFP:NbMIG21, or 35S-NbMIG21:RFP plasmid. Three days after agroinfiltration, confocal microscopy images of epidermal cells were obtained (Figure 5). A GFP fluorescence signal was detected in the cytoplasm, but the majority of the protein was localized to the nucleus, with a highest intensity in some subnuclear structures that likely correspond to the nucleolus. The same localization was observed for NbMIG21:RFP (Figure S3).

**Figure 6:**
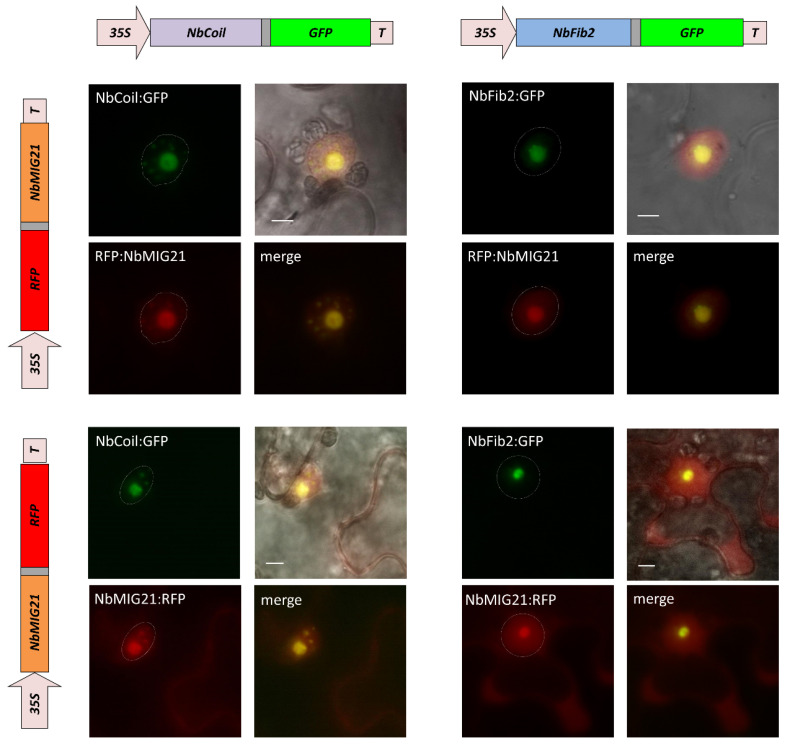
NbMIG21p co-localizes with fibrillarin and coilin. Schematic representation of genetic constructs encoding RFP-tagged NbMIG21p (**left**) and GFP-tagged fibrillarin or coilin (**top**). Fluorescent images of *N. benthamiana* cells 3 days after co-agroinfiltration with either 35S-NbMIG21:RFP or 35S-RFP:NbMIG21 and 35S-NbFib2:GFP or 35S-NbCoil:GFP. Nucleus is marked with a dashed line. Bar = 5 µm.

To further clarify the intranuclear localization of NbMIG21p, we obtained constructs encoding fibrillarin 2 (NbFib2) and coilin (NbCoil) fused with GFP. Fibrillarin is generally regarded as a nucleolus and Cajal body marker [35], and coilin is a major protein of Cajal bodies [36]. We co-infiltrated *N. benthamiana* leaves with agrobacterium containing 35S-NbFib2:GFP or 35S-NbCoil:GFP and agrobacterium with either 35S-RFP:NbMIG21 or 35S- NbMIG21:RFP plasmid. The RFP-tagged NbMIG21p fluorescence partly overlapped with GFP-tagged NbFib2 and NbCoil, indicating nucleolus and Cajal body localizations (Figure 6).

**Figure 7:**
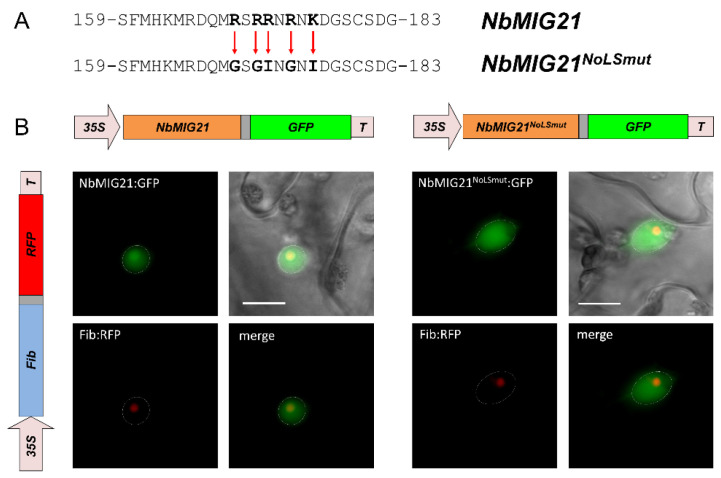
Mutagenesis of predicted NbMIG21p NoLS. (**A**) Schematic representation of mutations introduced in potential NbMIG21p NoLS sequence. (**B**) Fluorescent images of *N. benthamiana* cells 3 days after co-agroinfiltration with either 35S-NbMIG21:GFP or 35S-NbMIG21^NoLSmut^:GFP and 35S-NbFib2:RFP. Nucleus is marked with a dashed line. Bar = 10 µm.

According to the prediction, NbMIG21p contains an NoLS between 159 and 183 aa (Figure 4A). We substituted positively charged amino acid residues within this sequence with glycine and isoleucine (Figure 7A) and obtained the construct 35S-NbMIG21^NoLSmut^:GFP. A fluorescence microscopy analysis of the leaves co-agroinfiltrated with this construct and 35S-NbFib2:RFP revealed no GFP present in the nucleolus (Figure 7B), indicating that the predicted sequence indeed contains an NoLS, and lysine and arginine residues are responsible for its functioning.

**Figure 8:**
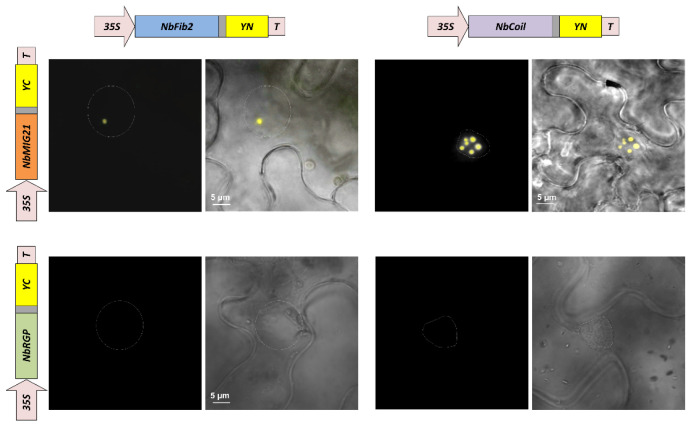
NbMIG21p colocalizes with fibrillarin and coilin. YFP fluorescence analyzed using fluorescent microscopy 3 days after infiltration of *N. benthamiana* leaves with pairs of agrobacteria containing plasmids for the expression of 35S-NbMIG21:YC and 35S-NbFib2:YN (**left**), 35S-NbMIG21:YC and 35S-NbCoil:YN (**right**). For each pair, a fluorescence image and superimposed-on-visible-light image are presented. 35S-NbRGP1:YC is used as a negative control. Nucleus is marked with a dashed line. Bar = 5 µm.

For the NbMIG21 intracellular distribution analysis, we also used a bimolecular fluorescence complementation (BiFC) system, in which analyzed proteins are fused to non-fluorescent N- (YN) or C-terminal (YC) fragments of yellow fluorescent protein (YFP) and co-expressed in the same cell. If the proteins interact or are localized in a distance less than 100 Å, a recovery of YFP fluorescence is observed. We generated constructs encoding NbMIG21p, NbFib2, and NbCoil fused with either YN or YC fragments of YFP. Pairs of constructs containing NbMIG21 and NbFib2 or an NbCoil sequence with a corresponding YFP fragment were co-expressed in *N. benthamiana* leaves. At 3 dpi, we observed YFP fluorescence for all pairs (Figure 8). For NbFib2:YN and NbMIG21:YC pair (or the opposite combination, NbFib2:YC and NbMIG21:YN), fluorescence was detected mainly in the nucleolus (Figure 8, left). For NbCoil:YN and NbMIG21:YC pair, YFP fluorescence was distributed in multiple subnuclear structures that corresponded to Cajal bodies (Figure 8, right). An antiviral protein, *N. benthamiana* reversibly glycosylated polypeptide 1 (NbRGP1) [37], was used as a negative control for the BiFC assay.

We concluded that NbMIG21p is distributed between the nucleolus and Cajal bodies. Moreover, the obtained results could indicate NbMIG21p interactions with NbFib2 and NbCoil.

### 2.5. NbMIG21 Interferes with Nucleocytoplasmic Transport

**Figure 9:**
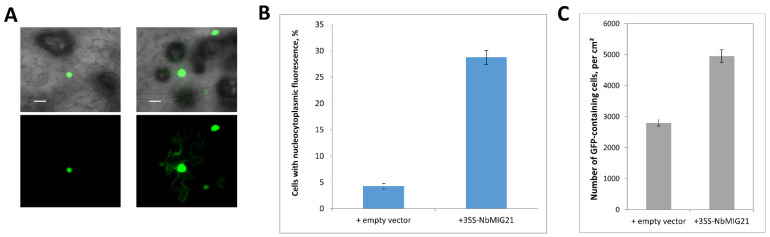
Increased expression of *NbMIG21* interferes with GFP:NLS nuclear import. (**A**) Representative images of nuclear (left) and nucleocytoplasmic (right) GFP:NLS distribution. Fluorescent image (lower panel) and overlay on bright-field image (upper panel). Bar = 20 µm. (**B**) Quantification of GFP:NLS subcellular localization 48 h after infiltration with 35S-GFP:NLS and 35S-NbMIG21 or “empty” vector. (**C**) Number of GFP:NLS-containing cells per square cm.

NbMIG21 was initially identified among methanol-inducible genes, and it shares some feature of these genes such as the ability to stimulate intercellular transport of macromolecules and facilitate tobacco mosaic virus reproduction [18]. Moreover, it has nuclear localization. Thus, we suggested that it could affect nucleocytoplasmic transport, as it was shown for PME [22] and for NbAELP [21]. To assess the efficiency of nucleocytoplasmic transport, we used a GFP reporter fused to human prothymosin alpha NLS. Normally, GFP:NLS localizes to the nucleus, but when co-expressed with NbMIG21, its distribution changes from nuclear to nucleocytoplasmic due to impeded nuclear import (Figure 9A). We quantified the percent of cells with GFP:NLS nucleocytoplasmic localization from the total number of cells with GFP fluorescence: in areas infiltrated with 35S-NbMIG21, this ratio increased ~5-fold compared to the control area (Figure 9B). These results indicate that elevated NbMIG21 production interferes with the nuclear import of NLS-containing proteins, partly retaining them in the cytoplasm. In addition, *NbMIG21* overexpression leads to an ~2-fold increase in the number of cells containing GFP:NLS compared to the control (Figure 9C). It could indicate either a more efficient transformation or more active intercellular transport, due to which fluorescence is observed not only in transfected cells, but also in neighboring cells, where GFP:NLS gets through the PD.

### 2.6. NbMIG21 Facilitates Viral Intercellular Transport

**Figure 10:**
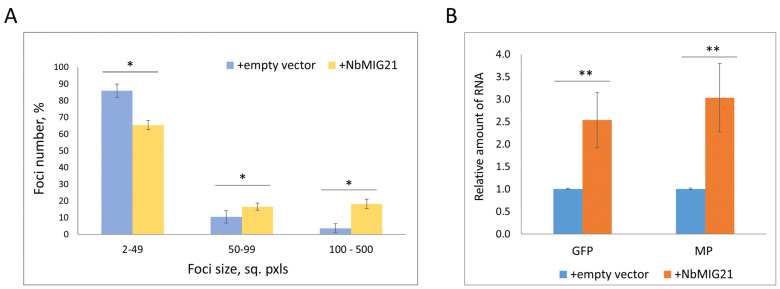
Increased *NbMIG21* expression stimulates TMV:GFP intercellular transport and reproduction. (**A**) The percentage of TMV:GFP-expressing foci of different sizes quantified at 5th day after agroinfiltration with TMV:GFP and “empty” vector or 35S-NbMIG21. (**B**) Relative amount of viral RNA as defined by qRT-PCR. *: *p* < 0.05; **: *p* < 0.01 (Student’s *t*-test).

Earlier, *NbMIG21* elevated expression was demonstrated to stimulate TMV-based GFP-encoding viral vector reproduction that was assessed by the quantification of GFP fluorescence [18]. However, the observed increase in GFP accumulation could be due to a more efficient viral RNA synthesis, stability, and enhanced intercellular transport. To distinguish between these processes, here, we assessed TMV:GFP cell-to-cell spread by measuring the foci sizes. For this, we used an optimized dilution of a TMV:GFP vector-containing *Agrobacterium* suspension to obtain individual infected cells. We performed a joint infiltration of *N. benthamiana* leaves with TMV:GFP and 35S-NbMIG21, while a combination with the “empty” vector was used as a negative control. Five days after infiltration, we quantified the ratio of GFP-containing foci of different sizes (Figure 10A) and viral RNA accumulation (Figure 10B).

The percentage of small foci (2–49 sq. pxls) decreased in areas with an elevated *NbMIG21* expression, while the number of larger foci (100–500 sq. pxls) increased there. The foci sizes reflect the efficiency of viral cell-to-cell movement, because each focus is the result of viral vector spread to adjacent cells from the initially infected cell followed by replication there. We also assessed the level of viral GFP- and movement protein (MP)-encoding RNA accumulation using qRT-PCR (Figure 10B) and showed that an increased *NbMIG21* expression leads to the significant stimulation of viral RNA levels. Thus, the obtained results indicate that TMV:GFP intercellular spread and RNA accumulation are more efficient in areas with an increased *NbMIG21* expression.

### 2.7. NbMIG21 Interacts with Promoter Regions In Vitro

**Figure 11:**
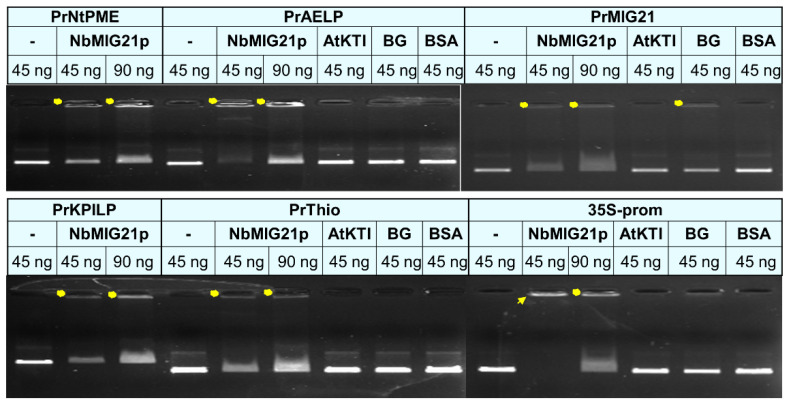
Gel retardation assay of PCR fragments representing promoter regions and 6xHis-NbMIG21. Two amounts of PCR fragments were used—45 and 90 ng—as indicated above each lane. NbMIG21p and control proteins were used in a concentration of 200 ng. Yellow dots indicate retarded NbMIG21p-bound PCR fragments; arrow indicates fully bound PCR fragment of 35S promoter. AtKTI: *A. thaliana* Kunitz trypsin inhibitor; BG: *N. benthamiana* beta-1,3-glucanase; and BSA: bovine serum albumin, were used as negative controls.

The nuclear and nucleolus localization of NbMIG21p may indicate its potential ability to interact with nucleic acids. To test this assumption, we obtained recombinant 6xHis-NbMIG21 in *Escherichia coli* cells and performed in vitro binding of this protein with different promoter regions, analyzing the results using a gel retardation assay (Figure 11). We used sequences of several available promoters of various *N. benthamiana* genes, including PrKPILP [38], PrThio [39], PrNtPME, and PrAELP [21], cauliflower mosaic virus 35S promoter and identified in this work PrMIG21, expecting that promoters contain common regulatory elements, which may be important for NbMIG21p binding.

Recombinant NbMIG21p was shown to interact both with plant gene promoters and a promoter from the phytovirus genome; NbMIG21p partly retains each tested DNA in the gel wells. Bovine serum albumin (BSA) and two recombinant plant proteins—*N. benthamiana* beta-1,3-glucanase (BG) [21] and *Arabidopsis thaliana* Kunitz trypsin inhibitor (AtKTI) [40]—were used as negative controls. Noteworthy, the best binding was observed for the 35S-promoter which correlates with this promoter’s efficiency compared to other tested sequences.

Based on the gel retardation assay results, we concluded that NbMIG21 is able to bind DNA in vitro.

## 3. Discussion

Gaseous methanol was previously demonstrated to be a signal molecule that activates MIGs expression. It was shown that both methanol treatment and the increased expression of such MIGs as *BG*, *NbAELP*, and *NbMIG21* stimulated the intercellular transport of 2xGFP and TMV-based viral vector reproduction [18]. However, the mechanism of MIGs activation and functioning is still to be elucidated. Here, we took a closer look at uncharacterized *NbMIG21*, identified earlier as a gene, the expression of which increases in response to gaseous methanol. The *NbMIG21*-encoded protein, NbMIG21p, is rich in glutamine residues, contains several amino acid repeats, and a predicted NLS and NoLS. We have found NbMIG21p homologues only among predicted proteins of *Solanaceae* species but not among annotated proteins. Noteworthy, all aligned sequences are rather conservative; they contain repeats and arginine/lysine- and serine-rich stretches in the C-terminal part (Figure 4).

The analysis of the NbMIG21p intracellular distribution revealed that it is indeed a nucleus- and nucleolus-localized protein. Lysine and arginine residues contained in the NoLS are essential for NbMIG21p nucleolus targeting, as was confirmed by mutagenesis. NbMIG21p potentially interacts with the major nucleolus protein fibrillarin and Cajal body protein coilin, as was shown in the co-localization experiments and using the BiFC system (Figure 8). Despite fibrillarin and coilin being regarded as markers of the abovementioned subnuclear structures, they are also detected in other regions of the nucleus. For example, RFP-tagged fibrillarin localized both to the nucleolus and Cajal bodies, while overexpressed coilin fused with GFP was visualized in the nucleoplasm, Cajal bodies, and nucleolus [41]. Thus, based on co-localization studies, we could conclude that NbMIG2p is distributed between the nucleoplasm, nucleolus, and Cajal bodies and might interact with fibrillarin and coilin. The nucleolus, besides being the site of rRNA synthesis and ribosome biogenesis, is also implicated in gene silencing, stress responses, and many other aspects of plant cell functioning [42]. Fibrillarin has multiple functions in plant cell among which is the participation in cell-to-cell and long-distance movements of viruses [43]. Coilin and Cajal bodies are also implicated in the responses to biotic and abiotic stress factors and serve targets for viruses [36,44]. Thus, NbMIG2p nucleolar localization and its interaction with both fibrillarin and coilin could indicate that these proteins are involved in the same stress-activated pathways. We could suggest that it serves the basis of the mechanism underlying methanol effects on plants in general.

Previously, it was shown that an increased *NbMIG21* expression stimulates the accumulation of GFP produced from the TMV-based viral vector [18]. However, viral vector reproduction is the sum of replication, RNA stability, and transport, and from the reported results, it was unclear whether the obtained effect was due to the facilitation of transport or the stimulation of viral RNA replication and accumulation. Here, we have shown that increased *NbMIG21* expression stimulated viral RNA accumulation and induced activation of local TMV:GFP spread, leading to the formation of larger GFP-expressing infection foci (Figure 10). Thus, conditions favorable for viral infection are created as a result of increased efficiency of local viral spread upon upregulated *NbMIG21* expression.

However, we could not exclude the impact of NbMIG21p’s effect on nucleocytoplasmic transport in the stimulation of viral vector reproduction. It is known that plant viruses of different taxonomy groups (including those viruses whose lifecycle takes place in the cytoplasm) exploit the nucleus of the host cell and interact with nuclear factors to facilitate infection [45]. Moreover, the same nuclear protein could play an opposite role for different viruses, by stimulating or suppressing it, as was shown, for example, for coilin [41]. NbMIG21p interferes with nucleocytoplasmic transport similar to another MIG—NbAELP–that was also demonstrated to impede GFP:NLS nuclear import [21] and stimulate viral vector reproduction [18]. PME, which is not a MIG but is a key player in methanol production, represents another example of the factor that prevents NLS-containing proteins from entering the nucleus in favor of viral infection: a PME-induced inhibition of ALY nuclear import results in a substantial increase in viral reproduction [22,46]. Thus, we suggested that one of the mechanisms of NbMIG21p-mediated facilitation of viral infection is realized via interference with nucleocytoplasmic transport.

Taking into account that the motif search engine (https://www.genome.jp/tools/motif/, accessed on 28 April 2023) revealed a motif resembling ubinuclein conserved middle domain in the NbMIG21p sequence (Figure S2), we can speculate that NbMIG21p might be involved in chromatin reorganization processes caused by abiotic stress, as was demonstrated for ubinucleins [47]. The online resource http://www.ebi.ac.uk/thornton-srv/databases/pdbsum/ (accessed on 29 October 2023) found a list of various protein domains with a rather low similarity to NbMIG21p, even though among them were nucleic acid-binding ones: zinc finger Ran-binding domain-containing protein 2 (O95218) localized in the nucleus [48] and ADP-ribosylation factor-like protein 6-interacting protein 4 (Q66PJ3) involved in modulating alternative pre-mRNA splicing also localized in the nucleus and nucleolus [49]. Taken together, NbMIG21p localization in the nucleus and nucleolus could indicate nucleic acid binding properties. Indeed, NbMIG21p was shown to be able to bind nucleic acids, in particular, DNA, as was demonstrated in vitro in the gel retardation assay. We tested promoter regions and revealed the most efficient binding for the 35S-promoter of the cauliflower mosaic virus (Figure 11), which is the “strongest” of all assessed sequences (i.e., provides the highest level of mRNA accumulation). Such a DNA-binding property could indicate that NbMIG21p participates in the regulation of intercellular transport, affecting gene expression. Nucleic acid binding proteins are crucial for various biological processes including replication, transcription, and translation [50]. Among these proteins are transcription factors, chromatin remodelers, RNA-binding proteins, etc. The regulation of their genes involves complex mechanisms that control their expression at the transcriptional and post-transcriptional levels. Various regulatory elements, such as enhancers, promoters, and transcription factor binding sites, modulate the expression of these genes. Inducible promoters are often found in genes that need to be turned on or off rapidly in response to changes in the cellular environment [51]. Inducible promoters allow for the dynamic control of gene expression, enabling cells to adapt to various internal and external cues, including stress, developmental signals, or environmental changes. The activators of such promoters are often small molecules or proteins. We isolated and characterized the *NbMIG21* promoter, which was experimentally confirmed to be activated in response to gaseous methanol. PrMIG21-mediated *GFP* mRNA accumulation significantly increased after 3 h incubation with gaseous methanol and started to decrease, but still was elevated, after a 24 h methanol treatment (Figure 3). This is in line with previously obtained results on endogenous *NbMIG21* expression; earlier, it was shown that the *NbMIG21* mRNA level increased after a 3 h incubation in a desiccator with methanol vapors and peaked after 21 h of further plant incubations in normal conditions [18]. According to the bioinformatic analysis, PrMIG21 contains various regulatory elements including stress-responsive and hormone-sensitive elements (Table S1). Methanol could act directly on some unidentified sequence in PrMIG21 or indirectly via known transcription factors; however, this is the subject of further studies.

Collectively, our results on *NbMIG21* properties and functions indicate that it is one of the key players that is responsible for the manifestation of a methanol-induced response of the plant cell. Here, we focused on *NbMIG21* effect towards viral infection; however, the other important aspect of methanol action is the induction of antibacterial resistance. *NbMIG21* involvement in it could be exhibited at the level of nucleocytoplasmic transport control and gene expression regulation. It is known that bacterial pathogens exploit the host cell nucleus during colonization; they possess special proteins—nucleomodulins—that can enter the nucleus and affect host gene expression, interfering with the plant’s immune response; thus, facilitating bacterial reproduction [52]. Methanol-induced susceptibility to the viral infection is the reverse side of acquired resistance to bacteria [18]. It is not the only example of balancing between resistance and susceptibility to pathogens: recently, it was shown that the increased production of NLS-containing proteins (as a mimetic of nucleomodulins) in plant cells leads to the induction of γ-thionin, a factor of antibacterial defense response. But the side effect of this cellular reaction is sensitivity to the virus: viral vector reproduction is stimulated both upon a γ-thionin elevated expression and massive synthesis of foreign NLS-containing proteins. The exact mechanism of these effects is unknown, but it likely involves interference with nucleocytoplasmic transport [39].

Therefore, the potential role of *NbMIG21* in resistance to bacterial pathogens and the mechanism of NbMIG21p functioning are the subjects of further studies as well as the properties of other MIGs.

## 4. Materials and Methods

### 4.1. Plant Growth Conditions

Wild type *Nicotiana benthamiana* plants were grown in pots with a mixture of leafy soil, humus, peat, and sand under standard conditions in a temperature-controlled greenhouse at 25/18 °C with a day/night cycle of 16/8 h. The 6–7-week-old plants with 5–6 true leaves were used in the experiments unless otherwise specified.

### 4.2. Agroinfiltration

*Agrobacterium tumefaciens* (strain GV-3101) was grown at 28 °C on LB medium with appropriate antibiotics (50 µg/mL rifampicin, 25 µg/mL gentamicin, and 50 µg/mL kanamycin or carbenicillin depending on the plasmid). Agroinfiltration buffer containing 10 mM MES (pH 5.5) and 10 mM MgCl2 was added to an aliquot of an overnight culture of *A. tumefaciens* to dilute it to the OD_600_ 0.1 or 0.005 for TMV:GFP experiments. Mixtures for infiltration, except for TMV:GFP and GFP:NLS experiments, were supplemented with agrobacteria containing plasmid for the expression of the p19 silencing suppressor of tomato bushy stunt virus. Leaves of *N. benthamiana* plants were infiltrated with *Agrobacterium* suspension using a syringe without a needle.

### 4.3. Whole Plant Methanol Treatment

To show endogenous *NbMIG21* activation by methanol treatment, *N. benthamiana* plants were incubated for 18 h in a 20 L sealed desiccator containing a filter paper with a 200 µL drop of methanol on it. Methanol readily evaporated, and the physiological elevated concentrations were obtained as was demonstrated earlier [18].

For the assessment of PrMIG21 methanol sensitivity, fully expanded leaves of *N. benthamiana* were infiltrated with agrobacterium containing PrMIG21-GFP plasmid. At 24 hpi, samples were collected using a hole punch, and three plants were placed into a 20 L desiccator that contained a 200 µL droplet of methanol on filter paper and sealed for 24 h, while the remaining three plants were maintained under standard conditions. Subsequently, all plants were kept without methanol for an additional 24 h. Samples from leaves were collected at several time points: 24, 27, 48, and 72 hpi.

### 4.4. GFP, RFP, and YFP Imaging and Quantification

GFP and YFP fluorescence was visualized using an AxioVert 200M microscope (Carl Zeiss, Jena, Germany) equipped with an AxioCam MRc digital camera. The excitation/detection wavelength (i) for GFP and YFP was 487/525 nm; for RFP: 561/625 nm, respectively. Confocal microscopy was performed using a Nikon C2 confocal laser scanning microscope (Nikon, Tokyo, Japan). The intracellular distribution of fluorescent proteins was observed 72 h after infiltration.

### 4.5. Assessment of Local TMV:GFP Spread

Individual TMV:GFP foci were analyzed 5 days post inoculation. GFP fluorescence in the infected leaves was observed and imaged under a handheld UV source. The TMV:GFP foci area was measured using open-source ImageJ software, version 1.47v [53]. The total number of foci was taken as 100%. Three biological repeats were performed.

### 4.6. Genomic DNA Extraction

Genomic DNA was extracted from frozen in liquid nitrogen *N. benthamiana* leaves using the Diatom DNA Prep kit as per the manufacturer’s protocol (Galart-Diagnosticum, Moscow, Russia).

### 4.7. RNA Extraction and cDNA Synthesis

Total RNA was extracted from plant tissues using TriReagent (MRC, Houston, TX, USA). The RNA concentration was determined using a Nanodrop ND-1000 spectrophotometer (Isogen Life Sciences, Utrecht, The Netherlands). First-strand cDNA was synthesized using random hexamers, oligo-dT primer, and Superscript II reverse transcriptase (Thermo Fisher Scientific, Waltham, MA, USA).

### 4.8. Quantitative Real-Time PCR (qRT-PCR)

Quantitative real-time PCR was performed using the iCycler iQ real-time PCR detection system (Bio-Rad, Hercules, CA, USA). Target genes were amplified using specific primers and EvaGreen master mix (Syntol, Moscow, Russia), while reference genes were amplified using primers to the 18S rRNA gene and protein phosphatase 2A gene (PP2A) (Table S2). Each sample was run in triplicate, and a nontemplate control was included. At least five biological replicates were performed, and the qRT-PCR results were analyzed using the Pfaffl algorithm [54].

### 4.9. Plasmid Constructs

To obtain the NbMIG21 coding sequence, PCR using *N. benthamiana* cDNA as a template with the primers N-NbMIG21-Acc65I_f/C-NbMIG21-SalI_r was performed, and the PCR product was cloned into the pAL2-T plasmid (Evrogen, Moscow, Russia). To obtain a set of constructs encoding NbMIG21 fusions with various fluorescent proteins (35S-NbMIG21:GFP, 35S-NbMIG21:RFP, 35S-NbMIG21:YC, and NbMIG21:YN), a fragment encoding NbMIG21 without a stop codon and flanked by Acc65I/BamHI recognition sites was synthesized using the N-NbMIG21-Acc65I_f/N-NbMIG21-BamHI_r pair of primers. The NbMIG21 fragment was digested with Acc65I/BamHI and inserted into the pCambia-35S vector containing either a GFP-, RFP-, YN-, or YC-encoding sequence without a start codon [37], using the same sites. The NbMIG21^NoLSmut^:GFP fragment was obtained as a product of overlap PCR, and mutations were inserted using NbMIG21-NoLS-mut_f and NbMIG21-NoLS-mut_r primers. The resulting fragment was inserted into pCambia-35S via Acc65I/PstI sites.

To obtain 35S-GFP:NbMIG21, 35S-YN:NbMIG21, and 35S-YC:NbMIG21 plasmids, an NbMIG21 fragment without a start codon was synthesized using the C-NbMIG21-*BamH*I_f/C-NbMIG21-*Sal*I_r pair of primers. A corresponding fluorescent tag-encoding sequence without a stop codon was amplified using the GFP-Acc65I_f/GFP-BamHI_r, YN-Acc65I_f/YN-BamHI_r, and YC-Acc65I_f/YC-BamHI_r pairs of primers, respectively. The NbMIG21 fragment was digested with BamHI/SalI, and GFP, YN, and YC fragments were digested with Acc65I/BamHI. Each fragment encoding the corresponding fluorescent tag together with the NbMIG21 fragment was ligated into a modified pCambia-35S vector digested with Acc65I/SalI enzymes.

Sequences encoding coilin (NbCoil, GeneBank accession number MK903618.1) and fibrillarin (NbFib2, GeneBank accession number AM269909.1) without a stop codon were amplified from *N. benthamiana* cDNA using the NbCoilin-Acc65I_f/NbCoilin-BamHI_r and NbFib2-Acc65I_f/NbFib2-BamHI_r primer pairs. The algorithm for obtaining 35S-NbCoil:GFP, 35S-NbCoil:YN, 35S-NbFib2:GFP, 35S-NbFib2:YN, and 35S-NbFib2:YC was the same as for NbMIG21-encoding constructs.

To obtain a plasmid for NbMIG21 recombinant protein production in *Escherichia coli* cells, an NbMIG21-encoding fragment without a start codon was obtained with the 6H-NbMIG21-Acc65I_f/C-NbMIG21-SalI_r primer pair and Acc65I and SalI recognition sites at the ends of the resulting fragment. The PCR product was digested with the Acc65I-SalI restriction enzymes and cloned into the pQE-30 vector (QIAGEN, Venlo, The Netherlands) digested with Acc65I/SalI. Therefore, 6xHis-NbMIG21 was obtained.

### 4.10. Production of Recombinant MIG21 Protein

NbMIG21 with an N-terminal hexahistidine tag (6xHis) was obtained according to the protocol outlined in the QIAexpressionist™ handbook. *E. coli* (strain SG13009) was used for accumulation of the recombinant protein. 6xHis-NbMIG21 purification was performed using affinity chromatography on Ni-NTA agarose resin. Eluted from the Ni-NTA column, 6xHis-NbMIG21 was analyzed using SDS PAAG electrophoresis and dialyzed using dialysis tubing (Spectrum Laboratories Specpor 4, 12–14K MWCO). The initial dialysis buffer contained 10 mM Tris-HCl (pH 4.5), 2 M urea and 80 mM NaH_2_PO_4_, with a stepwise reduction of urea and NaH_2_PO_4_ content. Dialysis was performed for 2 h in each buffer and in an overnight incubation in the final buffer (10 mM Tris-HCl (pH 4.5) and 20 mM NaH_2_PO_4_) at +4 °C. Further samples were concentrated using Sephadex dry powder.

### 4.11. In Vitro DNA Binding and Gel Retardation Assay

A total of 200 ng of dialyzed recombinant 6xHis-MIG21 was added to 45 or 90 ng of DNA fragments corresponding to each of the tested promoter sequences. Binding was performed in the buffer containing 20 mM Tris-HCl pH 7.5, 1 mM DTT, 3 mM MgCl_2_, and 50 mM NaCl for 1 h on ice. Bovine serum albumin, recombinant *A. thaliana* Kunitz trypsin inhibitor (AtKTI), and *N. benthamiana* beta-1,3-glucanase (BG) were used as negative controls. Samples were then loaded to 1% agarose gel containing ethidium bromide, and electrophoretic analysis was performed [55].

### 4.12. Statistical Analysis

Statistical analysis involved the use of Student’s *t*-test [56]. The *y*-axis error bars in all histograms represent the standard error of the mean values.

## Data Availability

Data are contained within the article and Supplementary Materials.

## Supplementary Materials

The following supporting information can be downloaded at: https://www.mdpi.com/article/10.3390/plants13020279/s1, Figure S1: *NbMIG21* pattern of expression according to version 6 of the Gene Expression Atlas; Figure S2: NbMIG21p motif search; Figure S3: NbMIG21p:RFP intracellular localization. Table S1: PrMIG21 cis-acting elements analysis. Table S2: Oligonucleotides used for qRT-PCR and cloning. References [57,58,59,60,61,62] are cited in the Supplementary Materials.
