# Supplementary material for: Nicotiana benthamiana Methanol-Inducible Gene (MIG) 21 Encodes a Nucleolus-Localized Protein That Stimulates Viral Intercellular Transport and Downregulates Nuclear Import
Source: Plants (Basel). 2024 Jan 17;13(2):279. doi: 10.3390/plants13020279 (PMC10819229; doi:10.3390/plants13020279)
Supplement: Supplementary file 1 [file plants-13-00279-s001.zip › plants-2734724-supplementary.pdf]

Supplementary material

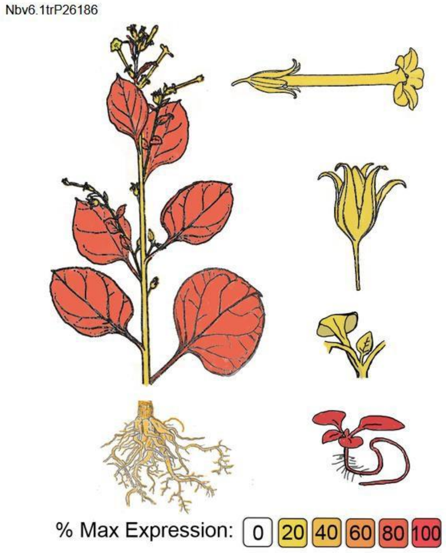

Figure S1. *NbMIG21* pattern of expression according to Version 6 Gene expression Atlas https://sefapps02.qut.edu.au/atlas/tREX6.php).

Pfam ID: UBN_AB etllkrvkklrlkeeeeklkellekLkeaidevmpeqiekYekevaalaeakaakka.........d

```
MASLQCHKPAQHAPSTLCQKTTTVTCNKANNEHHSFADKMKDMTDKMYHHDSHNHQSACHGTKTQQTAACHGTKTQQSAAC
HGTKTQQSAASHRTKTQQTACHGTSANGTKTQLSVACHGTKTQQSAASHGTKTQQTACHGTSATATHARACGKKKEGSFMH
KMRDQMRSRRNRNKDGSCSDGSDSSSSSSSDESDNENCGRTKNRGSC
```

Figure S2. Motif search service (https://www.genome.jp/tools/motif/) revealed that NbMIG21p fragment from 15 to 81 positions (red) contains motif corresponding to ubinuclein conserved middle domain (PF14075) (framed).

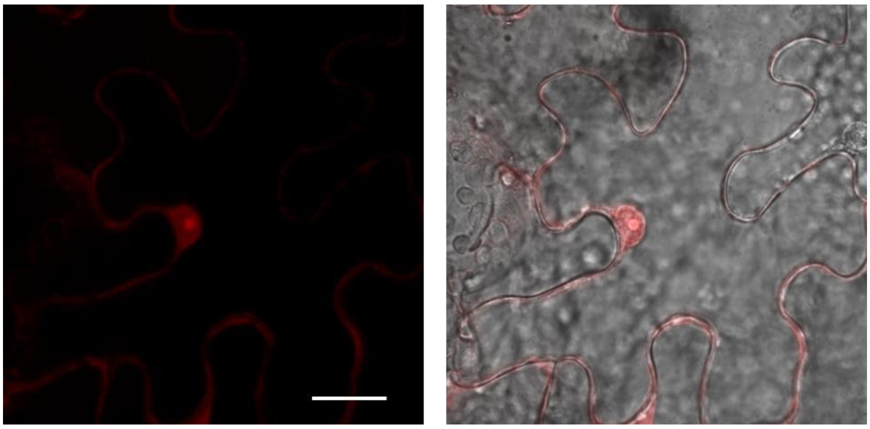

Figure S3. NbMIG21p:RFP intracellular localization. Images of 35S-NbMIG21:RFP expressing epidermal cells of *N. benthamiana* leaves 3 dpi obtained using confocal fluorescence microscopy. Projection of several confocal sections (left) superimposed on a bright field image of the same cell (right). Bar = 20 μm.

Table S1. PrMIG21 cis-acting elements analysis

Regulatory sequence	Position	Sequence	Function of site
**ABRE**	-1016	ACGTG	cis-acting element involved in the abscisic acid responsiveness
**ARE**	-944<br>-91	AAACCA<br>AAACCA	cis-acting regulatory element essential for the anaerobic induction
**Box 4**	-235	ATTAAT	part of a conserved DNA module involved in light responsiveness [57]
**MRE**	-704	AACCTAA	MYB binding site involved in light responsiveness
**MYB**	-767<br>-641	CAACCA	MYB-binding site [58]
**MYC**	-913<br>-607	CAATTG	MYC-binding site [59]
**MYB**	-801	TAACTG	MYB-binding site [60]
**TCA**	-561	TCATCTTCAT	salicylic acids response motif [61]
**STRE**	-1008	AGGGG	present in the promoters of stress-regulated genes and is recognized by the transcription factors Msn2p/4p [62]
**G-box**	-1016	CACGTT	cis-acting regulatory element involved in light responsiveness

Table S2. Oligonucleotides used for qRT-PCR and cloning

Name	Sequence
qRT-NbMIG21_f	GCAAATGGCACCAAAACTC
qRT-NbMIG21_r	GTAGCAGTGGCACTTGTTC
18S rRNA_f	ACGGCTACCACATCCAAG
18S rRNA_r	ACTCATTCCAATTACCAGACTC
PP2A_f	ATTGCTGCCTGTGGTTATTAC
PP2A_r	ATAGACTGAAGTGCTTGATTGG
N-NbMIG21-Acc65I_f	**GGTACC**ATGGCATCACTTCAGTGC
N-NbMIG21-BamHI_r	**GGATCC**GCAGCTCCCTCTATTC
C-NbMIG21-BamHI_f	**GGATCC**CTGGCATCACTTCAGTGC
C-NbMIG21-SalI_r	**GTCGAC**TCAGCTCCCTCTATTCTTG
NbMIG21-NoLS-mut_f	TGGGATCAACGGGAACATCGACGGAAG
NbMIG21-NoLS-mut_r	GTTCCCGTTGATCCCACTTCCCATTTG
GFP-Acc65I_f	**GGTACC**ATGAGCAAGGGCGAGG
GFP-BamHI_r	**GGATCC**TTTCTTGTACAGCTCGTCC
GFP-PstI_r	**CTGCAG**TTACTTGTACAGCTCGTCC
YN- Acc65I_f	**GGTACC**ATGGTGGTGAGCAAGGGCG

YN- BamHI_r	**GGATCC**CATGATATAGACGTTGTGGC
YC- Acc65I_f	**GGTACC**ATGGCCGACAAGCAGAAGAAC
YC- BamHI_r	**GGATCC**TTTGGACTTGTACAGCTC
NbCoilin-Acc65I_f	**GGTACC**ATGGAGGGCGTTAGGCTTC
NbCoilin-BamHI_r	**GGATCC**AATTTTGTTCTGGGATCTTAGG
NbFib2-Acc65I_f	**GGTACC**ATGGTTGCACCAACTAGAGG
NbFib2-BamHI_r	**GGATCC**GGCAGCAGCCTTCTGCTTC
6H-NbMIG21-Acc65I_f	**GGTACC**GCATCACTTCAGTGCC
PrNtPME-HindIII_f	**AAGCTT**GCTAGCAGGAACTAATCAGG
PrNtPME- Acc65I_r	**GGTACC**GAGCTCCCGGCGAAGAAATC
PrMIG21-HindIII_f	**AAGCTT**GTTACCATGTCTATGTGGAGC
PrMIG21-NruI_r	**TCGCGA**TGAATATGTCTGAAACTGAC
PrAELP-HindIII_f	**AAGCTT**AAAAACCTAACAATCC
PrAELP-NcoI_r	**CCATGG**CTCTAGTTGTTTTAGAG
PrKPILP-HindIII_f	**AAGCTT**AAATGAGAATTTACTTAAG
PrKPILP-NcoI_r	**CCATGG**TGTTAAGGATATGGTTAATG
PrThio-HindIII_f	**AAGCTT**ACCAGCACCTAAGC
PrThio-NcoI_r	**CCATGG**AGTTACTTTGAATGAGTAAAAAAG
